# YTHDF1 in Tumor Cell Metabolism: An Updated Review

**DOI:** 10.3390/molecules29010140

**Published:** 2023-12-26

**Authors:** Haichuan Rong, Danyang Wang, Yiran Wang, Chenshuang Dong, Guiling Wang

**Affiliations:** Key Laboratory of Cell Biology, Department of Cell Biology, Ministry of Public Health and Key Laboratory of Medical Cell Biology, Ministry of Education, China Medical University, Shenyang 110122, China; 2021340807@cmu.edu.cn (H.R.); wdy2330129794@163.com (D.W.); yrzy6666@163.com (Y.W.); 17302453686@163.com (C.D.)

**Keywords:** N6-methyladenosine, YTHDF1, metabolic reprogramming, cancer cell, molecular mechanisms

## Abstract

With the advancement of research on m6A-related mechanisms in recent years, the YTHDF protein family within m6A readers has garnered significant attention. Among them, YTHDF1 serves as a pivotal member, playing a crucial role in protein translation, tumor proliferation, metabolic reprogramming of various tumor cells, and immune evasion. In addition, YTHDF1 also exerts regulatory effects on tumors through multiple signaling pathways, and numerous studies have confirmed its ability to assist in the reprogramming of the tumor cell-related metabolic processes. The focus of research on YTHDF1 has shifted in recent years from its m6A-recognition and -modification function to the molecular mechanisms by which it regulates tumor progression, particularly by exploring the regulatory factors that interact with YTHDF1 upstream and downstream. In this review, we elucidate the latest signaling pathway mechanisms of YTHDF1 in various tumor cells, with a special emphasis on its distinctive characteristics in tumor cell metabolic reprogramming. Furthermore, we summarize the latest pathological and physiological processes involving YTHDF1 in tumor cells, and analyze potential therapeutic approaches that utilize YTHDF1. We believe that YTHDF1 represents a highly promising target for future tumor treatments and a novel tumor biomarker.

## 1. Introduction

The study of N6-methyladenosine (m6A) has garnered increasing attention in recent years, yielding remarkable achievements in various domains. m6A modification is a prevalent and vital epigenetic process in eukaryotic mRNA, signifying the methylation occurring at the N6 position of adenine nucleotides [1]. This modification serves to regulate the functionality of transcriptomes, ultimately impacting mRNA stability during translation. It plays a pivotal role in both development and disease progression within the organism [2,3]. m6A modification is a dynamic process comprising three distinctive components, namely, the “writers”, “erasers” and “readers” [4,5]. Writers, also known as methyltransferases, are responsible for catalyzing the m6A modification [6,7,8]. Erasers serve as demethylases responsible for the removal of methyl groups, thereby reversing the methylation process [9,10]. Readers predominantly encompass IGF2BP1, IGF2BP2, IGF2BP3, YTHDC1, and HNRNPA2B1 within the nucleus, along with YTHDF1, YTHDF2, YTHDF3, and YTHDC2 within the cytoplasm. These proteins act as m6A reader molecules, discerning and interpreting the methylation marks within cellular RNA [11,12,13]. Their primary function is to recognize the m6A modification sites on their downstream target genes, enabling m6A-modified RNA to exert specific effects and generate diverse biological responses. These effects encompass RNA translation, stability, processing, transport, and degradation [14,15,16,17,18].

**Figure 1:**
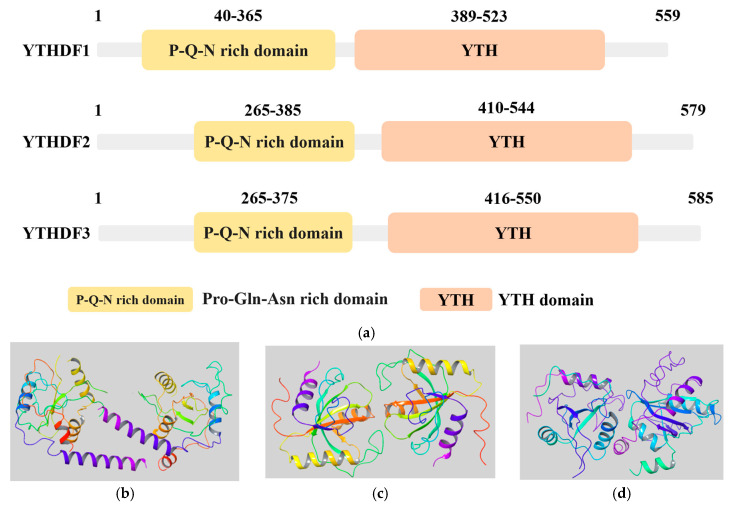
The structure of the YTHDF protein family. (**a**) Information on the YTH domains from the UniProt database (https://www.uniprot.org/, accessed on 15 November 2023). The YTH domain of the three variants of YTHDF proteins: YTHDF1 (UniProt ID: Q9BYJ9), YTHDF2 (UniProt ID: Q9Y5A9), YTHDF3 (UniProt ID: Q7Z739); (**b**–**d**) 3D structures of m6A reader YTHDF family proteins from RSCB PDB database (https://www.rcsb.org/, accessed on 15 November 2023). Non-protein molecules have been removed, leaving only the protein structure; (**b**) YTHDF1 (PDB ID: 4RCI); (**c**) YTHDF2 (PDB ID: 7Z8X); (**d**) YTHDF3 (PDB ID: 6ZOT).

The YTHDF family is currently the most prominent group of m6A “readers”. The YTHDF protein family comprises proteins containing the YTH domain (Figure 1a), including YTHDF1 (Figure 1b), YTHDF2 (Figure 1c), and YTHDF3 (Figure 1d). These proteins regulate the expression of downstream molecules by modulating the translation and stability of target mRNAs, thereby exerting influence on various physiological processes in organisms [19]. It is commonly perceived that the functionalities of YTHDF1/2/3 differ significantly from one another within their protein family. In comparison to other proteins in the family, YTHDF1 often assumes the role of facilitating the translation of m6A-mRNAs [19]. In contrast to YTHDF2, YTHDF1 is capable of earlier association with mRNAs throughout their lifecycle, thereby promoting translation [16]. Moreover, YTHDF1 collaborates with YTHDF3 to enhance translation [20]. Conversely, YTHDF2 primarily governs the stability of m6A-RNAs and the degradation of targeted m6A-mRNAs [21]. YTHDF3, on the other hand, exerts a bidirectional influence on mRNA translation and degradation by regulating YTHDF1 and YTHDF2 activity [22]. In recent years, there has been significant controversy surrounding this area of research. Some researchers have discovered that the YTHDF family exhibits conserved amino acids in close proximity to m6A recognition. Their mechanisms of m6A recognition and binding are completely identical, and all three proteins enact a collaborative activity that leads to the degradation of m6A-modified mRNA [23]. This suggests that the various proteins within the YTHDF family have interchangeable functions. They are capable of mutually assuming each other’s roles, but only when it comes to the degradation of m6A-mRNA do they act together. However, some researchers have countered this viewpoint, suggesting that while the depletion of the three YTHDF proteins does indeed result in increased stability of m6A-mRNA, this effect may not solely be related to the m6A function of YTHDF proteins. The overall decrease in YTHDF protein levels could potentially lead to mRNA exposure and the formation of P-bodies, which can enhance the stability of the mRNA system [24]. In conclusion, the role of the YTHDF protein family in cells and tumors continues to be worthy of further investigation.

Furthermore, a mounting body of research indicates that YTHDF1 not only plays a pivotal role in protein translation and biological growth processes, but also exerts critical influence within tumor cells. It is often upregulated in tumor cells, harnessing its remarkable m6A recognition and modification functions to mediate or regulate upstream and downstream factors, thereby driving the growth, metastasis, and metabolic reprogramming of cancer cells [25,26]. Moreover, the heightened expression of YTHDF1 exerts a tangible impact on tumor cells during therapeutic interventions. Hence, we contend that YTHDF1 can emerge as a novel participant in the oncological landscape, representing a promising therapeutic target with considerable potential. Moreover, it also holds tremendous prospects as a noteworthy biomarker.

However, there is a paucity of comprehensive reviews examining the mechanistic role of YTHDF1 in tumor cell metabolic reprogramming. In this review, we aim to present the latest comprehensive insights into the multifaceted involvement of YTHDF1, a member of the YTHDF family, in various aspects of tumor cell metabolism, with a particular focus on elucidating its unique characteristics in tumor cell metabolic reprogramming. Furthermore, we provide a comprehensive overview of the latest physiological and pathological processes involving YTHDF1 in tumors, with a special emphasis on investigating the potential mechanisms that could be harnessed for therapeutic interventions, thereby offering additional avenues of translational significance.

## 2. YTHDF1 and Tumorigenesis

**Figure 2:**
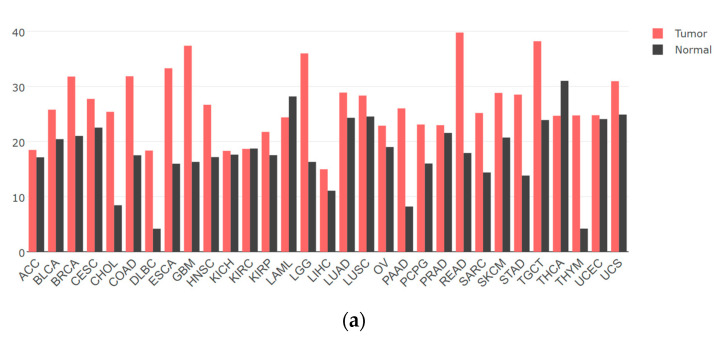

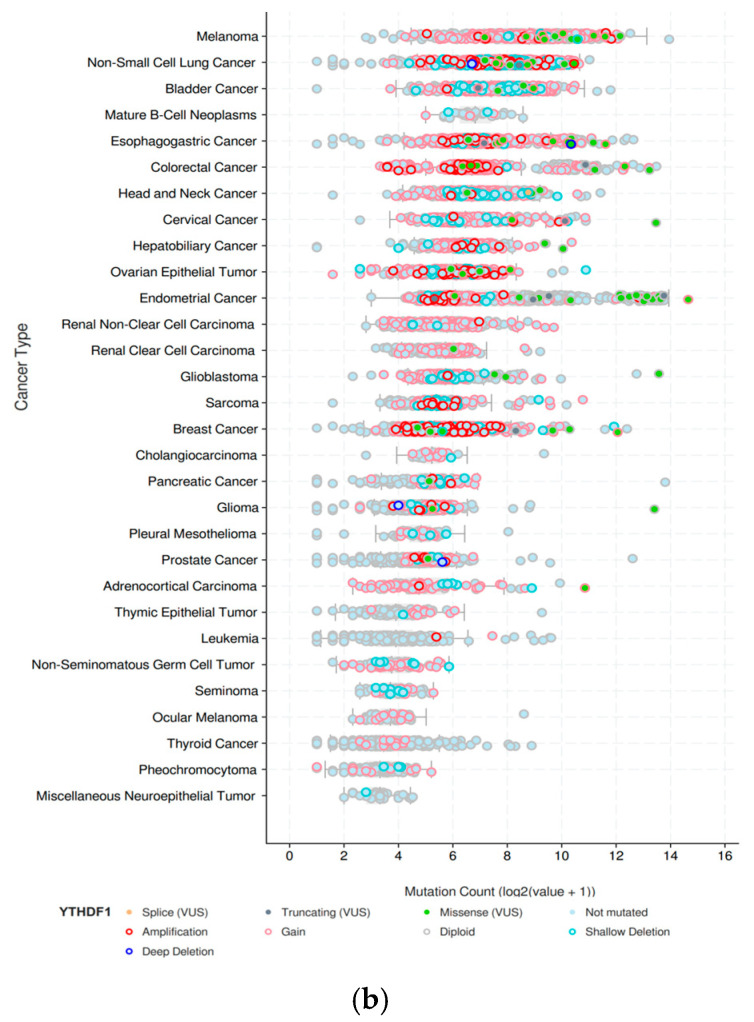
The gene expression and mutation of YTHDF1 in various types of tumors based on TCGA cancer data. (**a**) The expression profiles of the YTHDF1 gene in all tumor samples and their corresponding normal tissues derived using the GEPIA network tool (http://gepia.cancer-pku.cn/, accessed on 15 November 2023). The height of the bars represents the median expression level in each tumor type or normal tissue. (**b**) The mutation count of YTHDF1 in various types of tumors from cBioPortal database (https://www.cbioportal.org/, accessed on 15 November 2023). Each color of the dots represents a different mutation type. The vertical axis is sorted from top to bottom according to the median mutation count of each tumor type.

YTHDF1 is found to be highly expressed in the majority of tumors compared to normal tissues, exerting a promotional role in tumor development (Figure 2a). However, there have been relatively few studies on YTHDF1 protein mutations. Nevertheless, analysis of the TCGA database has revealed mutations in YTHDF1 across multiple tumor types (Figure 2b). Existing research suggests that these mutations may confer a higher risk of tumorigenesis, particularly in highly conserved amino acid residues of YTHDF1 [27]. In conclusion, abundant evidence has established a close association between YTHDF1 and tumor progression. Here, we summarize some of the recent findings on the mechanisms underlying the involvement of YTHDF1 in tumorigenesis.

### 2.1. Digestive System Tumors

Digestive system tumors continue to be a leading cause of cancer incidence and mortality worldwide. The most common digestive system tumors include colorectal cancer, gastric cancer, and liver cancer. In addition, other digestive system tumors include esophageal cancer and pancreatic cancer.

#### 2.1.1. Colorectal Cancer

Colorectal cancer (CRC) is the most prevalent and deadliest malignancy among gastrointestinal tumors. Current research overwhelmingly suggests that YTHDF1 is highly expressed in gastrointestinal tumors and is associated with poor prognosis, indicating its carcinogenic role [28]. The amplification of the YTHDF1 DNA copy number is a common event in cancer, leading to the overexpression of YTHDF1 in this disease [29]. YTHDF1 has been found to play a regulatory role in the occurrence and development of CRC through various novel metabolic pathways. As a nucleocytoplasmic protein, the cytoplasmic localization of YTHDF1 is dependent on O-GlcNAc (O-linked GlcNAc) glycosylation modification. It interacts with O-GlcNAc transferase (OGT), and O-GlcNAc facilitates the cytoplasmic localization of YTHDF1. The recruitment of YTHDF1 upregulates the expression of downstream target gene c-Myc. Overexpression of c-Myc has been established to promote tumor cell growth in multiple types of cancer, suggesting that the newly discovered OGT–YTHDF1–c-Myc axis is likely the foundation of CRC development [30].

#### 2.1.2. Gastric Cancer

The expression of YTHDF1 in GC patients is significantly elevated compared to normal tissues. YTHDF1 has been demonstrated to be a potential predictive marker in gastrointestinal tumors, exerting a significant impact on tumor occurrence, metastasis, and prognosis [31]. YTHDF1 has also been discovered to play a regulatory role in GC through its involvement in various newly discovered metabolic axes. Autophagy in GC has been found to promote disease progression, which is intricately linked to the m6A/YY1/ATG4B metabolic axis in which YTHDF1 participates. YTHDF1 can enhance the stability and expression of YY1 mRNA in an m6A-dependent manner. Furthermore, YY1 induces ATG4B-dependent cellular autophagy by binding to the ATG4B promoter, ultimately fostering the progression of GC [32]. Recently, a novel UCA1/miR-145-5p/YTHDF1 axis has been discovered, in which PLAGL2 (Pleiomorphic Adenoma Gene-Like 2) can bind to the upstream promoter of UCA1. This interaction induces a sponge effect of UCA1 on miR-145-5p, thereby regulating YTHDF1. Subsequently, YTHDF1 interacts with eEF-2, enhancing the m6A modification of Snail translation, ultimately leading to the epithelial–mesenchymal transition (EMT) of GC cells [33]. Under hypoxic conditions, YTHDF1 strongly promotes the progression of GC. HIF-1α (Hypoxia-Induced Factor-1α) binds to the promoter region of H19, inducing its overexpression. H19, through the m6A modification mediated by YTHDF1, leads to the overexpression of SCARB1, thereby promoting proliferation, migration, and angiogenesis in GC cells [34].

#### 2.1.3. Esophageal Cancer

The two main histological subtypes of esophageal cancer are adenocarcinoma and squamous cell carcinoma. Esophageal squamous cell carcinoma (ESCC) accounts for 85% of all esophageal cancer cases globally [35]. In the realm of ESCC, recent discoveries have unveiled the enrichment of microbiota communities, such as Fusobacterium nucleatum (Fn), which possess the ability to foster ESCC development. It is within this intricate landscape that YTHDF1 assumes a pivotal role. Fn, in its interactions, elevates the expression of METTL3, thereby inducing the methylation of the 3’-UTR of c-Myc mRNA via YTHDF1. This, in turn, fortifies the stability of c-Myc mRNA, culminating in a heightened translational activity that ultimately drives the growth and invasive characteristics of ESCC [36].

#### 2.1.4. Liver Cancer

Approximately 90% of primary liver cancer cases are hepatocellular carcinoma (HCC), making it the most common form of liver cancer [37]. The development and metastasis of HCC have been a subject of significant interest. YTHDF1, often upregulated in HCC, has emerged as a relevant prognostic indicator associated with this disease [38]. In the study on the bone metastasis of liver cancer, a recent report has highlighted the functional role of YTHDF1. YTHDF1 collaborates with METTL3 to promote the translation of Anillin actin-binding protein (ANLN) in an m6A-dependent manner. Subsequently, ANLN forms a complex with SP1 to facilitate the transcription and translation of downstream KIF2C. This activation of the mTORC1 signaling pathway is closely associated with the expression of RANKL and the dysregulation of RANKL-OPG within the bone microenvironment, representing an essential factor contributing to HCC bone metastasis. These findings suggest that YTHDF1 may serve as an upstream regulatory factor involved in HCC bone metastasis [39]. Furthermore, it has recently been demonstrated that YTHDF1 enhances the translation efficiency of epidermal growth factor receptor (EGFR) mRNA through its m6A reader function, thereby promoting the progression of intrahepatic cholangiocarcinoma [40].

#### 2.1.5. Pancreatic Cancer

Early symptoms of pancreatic cancer are often vague, making diagnosis challenging. With its extremely malignant nature and poor prognosis, pancreatic cancer ranks as the seventh leading cause of cancer-related deaths. Over 90% of pancreatic cancers are pancreatic ductal adenocarcinoma (PDAC) [41,42]. PDAC is a highly stromal-rich tumor, and the differential expression of RNA m6A modification, represented by YTHDF1, plays a significant role in prognostic evaluation in both tumor cells and stromal cells [43]. It has been observed that the newly discovered YTHDF1–GLS1 axis in PDAC can be disrupted by triterpenoid compounds with drug activity, such as olean-28,13β-lactam (B28), which inhibit the growth of pancreatic cancer. The mechanism behind this is that B28 reduces the expression of YTHDF1, consequently downregulating the KGA/GAC isoform of GLS1. The loss of GLS1 inhibits tumor cell viability, increases overall reactive oxygen species (ROS) levels, induces a bioenergetics crisis, promotes oxidative stress, and ultimately leads to tumor apoptosis [44].

### 2.2. Respiratory System Tumors

Malignant tumors commonly observed in the respiratory system often include lung cancer, nasopharyngeal cancer, laryngeal cancer, and others. Non-small cell lung cancer is particularly representative of lung cancer. The occurrence and progression of respiratory system cancers are critically influenced by the pivotal role of YTHDF1.

#### 2.2.1. Non-Small Cell Lung Cancer

Non-small cell lung cancer (NSCLC) is the predominant histological subtype of lung cancer, accounting for approximately 85% of all cases [45]. Currently, there are studies confirming that YTHDF1 in tissues can serve as a predictive indicator for NSCLC [46]. YTHDF1 functions as an m6A-dependent mediator in NSCLC, facilitating METTL3-mediated methylation modification of FRAS1 (Fraser extracellular matrix complex subunit 1) mRNA, thereby regulating the expression of CDON (cell adhesion-associated, oncogene-regulated). Both FRAS1 and CDON are involved in tumor progression, promoting tumor cell proliferation and colony formation [47]. YTHDF1 collaborates with YTHDF3 to specifically recognize TGFβR2 (TGF-β receptor 2) and SMAD3 (SMAD Family Member 3) mRNA, thereby facilitating the promotion of TGF-β-induced epithelial–mesenchymal transition (EMT) leading to NSCLC progression. However, this recognition and binding process is counteracted by the intervention of ALKBH5 [48].

#### 2.2.2. Nasopharyngeal Carcinoma

As it is widely known, the occurrence of nasopharyngeal carcinoma (NPC) is often associated with the presence of Epstein–Barr virus (EBV) infection [49]. EBV primarily infects B cells and epithelial cells, leading to their malignant transformation and triggering the development of tumors. Recent discoveries have unveiled that YTHDF1 can regulate the transcriptional activation pathway associated with EBV. YTHDF1 coordinates with ZAP, DX17, and RNA decapping enzyme DCP2 to decap and decrease the stability of EBV activator BZLF1 mRNA and BRLF1 mRNA. BZLF1 and BRLF1 play vital roles in the reactivation and lytic replication of the virus [50]. The inhibition of these factors also signifies the suppression of EBV activation and reinfection, ultimately restraining the occurrence of NPC [51].

#### 2.2.3. Laryngeal Cancer

Research on the involvement of YTHDF1 in laryngeal cancer (LC) is relatively limited. However, a recent study by Li has reported the latest findings on the association between YTHDF1 and cancer-associated fibroblasts (CAFs) in LC. CAFs are important constituents of the tumor microenvironment, and YTHDF1 may be involved in their functions. The researchers discovered the presence of LncRNA TUC338 in extracellular vesicles (EVs) released by CAFs, which are involved in communication with surrounding cells. This LncRNA TUC338 was found to regulate the invasiveness of LC through the miR-8485/CBX2 axis. YTHDF1, in conjunction with METTL3, appears to play a role in stabilizing and regulating the translational expression of this critical factor, LncRNA TUC338. However, the specific mechanisms by which YTHDF1 and METTL3 impact LC have not been fully investigated [52]. In summary, the specific mechanisms by which YTHDF1 is involved in the occurrence and progression of LC are still unknown.

### 2.3. Genitourinary System Tumors

YTHDF1 plays a significant role in genitourinary cancers, particularly in kidney cancer, bladder cancer, common prostate cancer in males, as well as common breast cancer and cervical cancer in females.

#### 2.3.1. Renal Cell Carcinoma

Renal cell carcinoma (RCC) is the most common type of cancer in the genitourinary system. Radical nephrectomy or partial nephrectomy, aimed at localized renal tumors, has shown satisfactory therapeutic efficacy [53]. However, the role of YTHDF1 in the process of malignant metastasis of advanced RCC is significant, and understanding the specific mechanism of YTHDF1 in RCC is valuable. Zhang reported that YTHDF1 can be mediated by METTL14 overexpression, enhancing the stability of Pten mRNA in an m6A-dependent manner and thereby inhibiting the proliferation and migration of tumor cells, activating AKT signaling, and suppressing tumor progression [54]. Furthermore, another study suggests that YTHDF1 can enhance the expression level of ZNF677 (zinc finger protein 677) through m6A modification. Upregulated ZNF677 significantly inhibits CDKN3 (cyclin-dependent kinase inhibitor 3) at both mRNA and protein levels, inhibiting tumor growth and inducing cell apoptosis, exerting a negative impact on RCC progression [55].

#### 2.3.2. Bladder Cancer

Bladder cancer (BLCA) is one of the most common malignancies in the urinary system [56]. According to database analysis, YTHDF1 is highly expressed in BLCA tissues, and is closely associated with the clinical prognosis of BLCA patients such that the mechanism of YTHDF1 in BLCA is worth investigating [57]. A novel METTL3/YTHDF1-RPN2-PI3K/AKT/mTOR regulatory axis has been reported, where the upregulation of METTL3/YTHDF1 levels enhances the stability of RPN2’s (ribophorin II) mRNA and protein [58]. RPN2 may accelerate tumor cell proliferation and the progression of BLCA by activating the notorious PI3K/AKT pathway [59].

#### 2.3.3. Prostate Cancer

Prostate cancer (PRAD) is one of the most prevalent non-cutaneous malignancies in men worldwide. Despite the proven efficacy of androgen deprivation therapy (ADT) in improving overall survival rates of PRAD patients, subsequent tumors may develop resistance, leading to the progression of castration-resistant PRAD [60]. YTHDF1 plays a pivotal role in the tumorigenesis and progression of PRAD, and is associated with unfavorable prognosis. A recent study has revealed the critical involvement of the ELK1/YTHDF1/PLK1/PI3K/AKT axis in PRAD. YTHDF1 in PRAD is transcriptionally activated by ELK1 and exhibits high expression. The key factor, PLK1 (Polo-like kinase 1), is directly targeted for translation by YTHDF1 in an m6A-dependent manner during the cell cycle. The upregulation of PLK1 further promotes the progression of PRAD through the PI3K/AKT signaling pathway [61].

#### 2.3.4. Breast Cancer

Breast cancer (BRCA) has a high global incidence and ranks among the most common malignancies in women [41]. The survival rates for advanced-stage and recurrent/metastatic BRCA are poor [62]. YTHDF1 has been identified as a potential prognostic biomarker in the comprehensive analysis of BRCA [63]. m6A modification affects the activation, invasion, and immune escape of tumor-associated immune cells [64]. A recent study has indicated that YTHDF1 is highly expressed in BRCA tumor tissue, creating a “cold” tumor microenvironment by suppressing the release of pro-inflammatory cytokines Ccl7, Il-17rb, Sell, Cxcl2, and Tnfsf4 in BRCA cells. This ultimately hinders the infiltration of anti-tumor CD8+ T cells and impairs T cell functional differentiation, thereby promoting the immune escape of BRCA cells [65].

#### 2.3.5. Cervical Cancer

Cervical cancer (CC) ranks as the fourth most commonly diagnosed cancer and the fourth leading cause of cancer-related female mortality. Its recurrent and metastatic nature contributes to poor prognosis [41]. In recent immunological studies of CC, a risk signature based on prognostic features (METTL16, YTHDF1, and ZC3H13) has been identified as an independent prognostic indicator for CC. YTHDF1 exhibits a negative correlation with PD-L1 expression, and may serve as a crucial mediator regulating PD-L1 expression and the tumor immune microenvironment, significantly influencing the development of CC [66].

### 2.4. Other Tumors

YTHDF1 exhibits high expression in various types of tumors, and its mechanisms are quite diverse.

#### 2.4.1. Thyroid Cancer

Thyroid cancer (THCA) ranks ninth in terms of global cancer incidence, and its prevalence has been rapidly increasing in recent years [41]. It is the most common malignancy among adolescents and adults aged 16–33 [67]. The differential expression of m6A regulatory factors, represented by YTHDF1, has been observed in normal tissues and papillary thyroid carcinoma (PTC). According to recent research, METTL3 can increase the stability of STEAP2 mRNA (six transmembrane epithelial antigen of the prostate 2) in a YTHDF1-dependent manner. In PTC, however, the expression of METTL3 is reduced, resulting in the decreased YTHDF1-dependent translation of STEAP2 mRNA. The low expression of STEAP2 promotes tumor invasiveness and poor prognosis through the activation of the Hedgehog signaling pathway and epithelial–mesenchymal transition (EMT) [68].

#### 2.4.2. Osteosarcoma

In osteosarcoma, recent studies have revealed that YTHDF1 is positively correlated with the m6A-dependent translational enhancement of YAP (Yes-associated protein 1). Methylated YAP transcripts serve as potential targets of YTHDF1, and YTHDF1 recognizes methylated YAP transcripts to promote its translation. The aberrant downregulation of the demethylase ALKBH5 (ALKB homolog 5) in osteosarcoma leads to the upregulated expression of YAP, consequently enhancing the proliferation, invasion, and metastatic ability of osteosarcoma cells [69] (Table 1).

## 3. YTHDF1 and Cancer Metabolism

Tumor cell metabolic reprogramming is a survival strategy adopted by cancer cells to meet their nutritional and energy requirements. They actively modify their metabolic patterns to promote the growth and proliferation of tumors [70]. Multiple metabolic pathways play a role in tumor cell metabolic reprogramming, including glucose metabolism reprogramming, glutamine metabolism reprogramming, and macrophage metabolism reprogramming. Among these, YTHDF1 plays a crucial role.

### 3.1. Glucose Metabolism

Metabolic reprogramming of the glucose metabolism is a highly influential strategy in the realm of metabolic reprogramming. Tumor cells adeptly utilize this rewiring of the metabolism, transforming the traditional pathways of glycolysis, pentose phosphate pathway, serine pathway, and tricarboxylic acid cycle into a proficient means of rapidly generating ATP and other essential molecules to drive their own growth [71]. The Warburg effect stands as the quintessential hallmark of glucose metabolism reprogramming, enabling tumor cells to engage in glycolytic reactions to acquire ATP and other essential substances even in aerobic conditions, without relying on mitochondrial oxidative phosphorylation for energy production [72]. The m6A modification function possessed by YTHDF1 plays a considerable role in the metabolic reprogramming of glucose, particularly within the context of the Warburg effect.

YTHDF1 often interacts with other m6A-modifying proteins to facilitate the occurrence of the Warburg effect. In non-small-cell lung cancer, METTL3 can enhance the stability of long non-coding RNA DLGAP1-AS2 (DLGAP1 antisense RNA2) through its m6A reader function. Furthermore, YTHDF1 can interact with DLGAP1-AS2 in an m6A-dependent manner, thereby promoting the translation of c-Myc mRNA. As a crucial regulatory factor in the Warburg effect, the upregulation of c-Myc level can enhance the occurrence of the Warburg effect [73]. In Treg cells, recent research has revealed a collaborative regulation of c-Myc levels by YTHDF1 and the deubiquitinase USP47. USP47 can prevent the ubiquitination of YTHDF1 and reduce its ability to promote translation, thereby exerting control over c-Myc expression. If the level of USP47 decreases, c-Myc will be overexpressed and trigger the Warburg effect [74]. The m6A “eraser” protein FTO can also regulate c-Myc and collaborate with YTHDF1. The promoter of FTO can be downregulated by the Wnt/β-Catenin pathway. Significant downregulation of FTO can globally increase the methylation levels of metabolic genes’ mRNA, including c-Myc, and recruit YTHDF1, leading to the upregulated expression of c-Myc and promoting aerobic glycolysis in tumors [72]. YTHDF1 can also interact and collaborate with METTL3. The recruitment of the YTHDF1 protein and the m6A-dependent mechanism of METTL3 work together to promote the translation of LDHA (Lactate dehydrogenase A) mRNA, forming a METTL3/LDHA metabolic axis. The accumulation of LDHA directly promotes aerobic glycolysis, leading to the occurrence of the Warburg effect [75]. In cervical cancer, the expression level of HK2 (hexokinase 2) is also regulated by the collaborative action of YTHDF1 and METTL3. HK2 is one of the key enzymes in the Warburg effect. METTL3, through the recognition function of YTHDF1, can interact with HK2 mRNA, enhancing the expression of the HK2 protein, thus promoting the Warburg effect [76]. A similar mechanism also exists in esophageal cancer, where YTHDF1 can directly interact with HCP5 (HLA Complex P5), enhancing the interaction between YTHDF1 and HK2 mRNA to promote aerobic glycolysis [77]. In the context of cervical cancer, the translation of the PDK4 protein is also under the regulatory influence of YTHDF1. The interaction between YTHDF1 and eEF1 fosters the promotion of PDK4 mRNA translation. The excessive expression of PDK4 is intimately associated with the facilitation of aerobic glycolysis and tumor progression [78]. Within the course of lung adenocarcinoma (LUAD) genesis, there occurs an m6A-dependent aerobic glycolysis, wherein the pivotal determinant for m6A-dependent glycolysis is ENO1 (Enolase 1). The methylation of ENO1 mRNA displays a positive correlation with METTL3 but a negative correlation with ALKBH5. Methylated ENO1 mRNA can be recognized by YTHDF1, thus enhancing the stability of ENO1 mRNA. ENO1, in turn, facilitates the generation of phosphoenolpyruvate (PEP) through the catalysis of 2-phospho-D-glycerate (2-PGA), thereby emerging as a critical participant in promoting aerobic glycolysis and serving as a stimulus for tumor development pathways [79]. YTHDF1 can also cooperate with WTAP to stabilize FOXP3 mRNA. FOXP3 can interact with SMARCE1, activating and promoting the progression of the Warburg effect [80].

Hypoxic conditions commonly serve as prominent factors contributing to the initiation, progression, and dissemination of tumors, as is widely evident within solid neoplasms. Additionally, under hypoxic circumstances, the glycolytic reactions exhibit heightened activity, with YTHDF1 assuming a critical role in this process. Fascinatingly, recent investigations have revealed that miR-16-5p possesses the ability to disrupt the stability of YTHDF1 mRNA. Notably, the elevation of HIF-1α levels in hypoxic tumor cells suppresses the expression of miR-16-5p, facilitating the recruitment of YTHDF1 and its subsequent binding to the pivotal enzyme PKM2 mRNA within glycolysis. This interaction ensures the sustained expression of PKM2, ultimately promoting the occurrence of glycolysis [81].

### 3.2. Glutamine Metabolism

In addition to the pivotal role played by glucose metabolism in the metabolic reprogramming of tumors, the reprogramming of glutamine metabolism also holds significant importance that cannot be ignored. Glutamine, being one of the most abundant amino acids in the human body, serves as a crucial source of nitrogen [82]. Glutamine provides the essential energy required for the autonomous growth and proliferation of tumor cells. Additionally, the reprogramming of glutamine metabolism is closely linked to the tumor’s immune evasion ability within the tumor microenvironment [83,84]. Thus, tumor cells exhibit a pronounced increase in the demand for glutamine compared to normal cells in order to fulfill their survival requirements. Tumor cells utilize specific transport proteins, such as SLC1A5 or ASCT2, to efficiently transport glutamine into the cell. Subsequently, through the action of GLS, glutamine is enzymatically converted into glutamate [85], which further transforms into α-ketoglutarate. This metabolic pathway enables tumor cells to generate energy through the tricarboxylic acid cycle, thereby facilitating tumor growth and metastasis [86,87]. YTHDF1, as an upstream gene of GLS, is closely associated with the reprogramming of glutamine metabolism in tumor cells. YTHDF1 directly interacts with GLS1 mRNA to facilitate the translation of GLS1. GLS1, as a crucial component of glutaminase, plays an irreplaceable role in mediating glutamine metabolism. Studies have shown that GLS1 is upregulated in tumors, indicating its close relationship with the role played by YTHDF1 in tumorigenesis [88].

### 3.3. Macrophage Metabolism

Macrophage Metabolism Reprogramming is an essential aspect of tumor metabolism reprogramming. Macrophages can be classified into two distinct categories, namely, M1 and M2, representing classically activated macrophages and alternatively activated macrophages, respectively. These two cell types exhibit almost contrasting roles [89]. Tumor-associated macrophages (TAMs) play a crucial role in the tumor microenvironment, influencing tumor initiation, proliferation, invasion, and metastasis [90]. YTHDF1 also exerts its effects in tumor-associated macrophages.

The secretion of the chemokine CXCL16 by TAMs is closely associated with the impact of m6A levels in tumors. CXCL16 can interact with its receptor CXCR6, not only upregulating CXCR6 expression but also increasing m6A levels within tumors. This, in turn, leads to the upregulation of YTHDF1 and WTAP levels, while downregulating ALKBH5 expression. These changes collectively promote tumor invasion and drug resistance [91]. YTHDF1 also collaborates with METTL3 in macrophage metabolism. METTL3 regulates macrophage polarization towards M1 and M2 phenotypes through the NF-κB and STAT3 signaling pathways. Furthermore, during this process, METTL3 simultaneously modulates the YTHDF1-mediated translation of SPRED2 mRNA. The expression levels of SPRED2 in macrophages are closely associated with the phosphorylation changes of ERK, NF-κB, and STAT3, all of which have significant impacts on macrophage metabolic reprogramming and tumor growth and metastasis [92].

### 3.4. Iron Metabolism

Iron is an essential element for tumor growth and proliferation, and its functions in oxidation-reduction and the generation of free radicals play crucial roles in tumor formation and growth. The acquisition, utilization, and excretion of iron are all influenced by tumors, indicating a close connection between iron metabolism reprogramming and tumorigenesis [93]. With continuous advancements in ferroptosis research, the crucial role of YTHDF1 in tumor iron metabolism has garnered significant attention.

YTHDF1 exerts its regulatory role in tumor iron metabolism through various signaling pathways by modulating the functions associated with m6A modification. It directly interacts with the mRNA of TFRC (Transferrin Receptor Protein 1), one of the most crucial proteins in iron metabolism, promoting the upregulation of TFRC translation. This leads to an increase in cellular iron content, Fe^2+^ levels, and ROS concentration, thereby driving tumor cell occurrence, proliferation, and invasion [94]. YTHDF1 is intimately involved in iron metabolism related to ferroptosis. It binds to CircSAV1, forming a ternary complex that acts on IREB2 mRNA. This promotes the translation of IREB2, and elevated levels of IREB2 result in iron accumulation and lipid peroxidation, which are essential conditions triggering ferroptosis. Therefore, CircSAV1 serves as one of the mediators of ferroptosis, exerting its effects through YTHDF1 [95]. YTHDF1 also contributes to the enrichment of FTH (Ferritin H) mRNA and promotes translation in an m6A-dependent manner. FTH is considered an indispensable component of iron metabolism, playing a crucial role in the cellular processes of iron uptake and storage. The disruption of iron metabolism can lead to the outcome of ferroptosis. YTHDF1 shows a positive correlation with FTH levels, and is likewise positively associated with GPX4, a key regulator of ferroptosis. This suggests that YTHDF1 plays a significant role in the iron metabolism involved in [96].

However, some studies have found contradictory conclusions. In prostate cancer, the overexpression of YTHDF1 often inhibits tumor ferroptosis. After YTHDF1 overexpression, intracellular levels of Fe2+, malondialdehyde, and ROS decrease, while the level of glutathione (GSH) increases, significantly suppressing ferroptosis. This may be due to the fact that YTHDF1 overexpression helps tumors evade immune attacks mediated by CD8+ T cells and ferroptosis [97].

### 3.5. Other Metabolisms

YTHDF1, serving as a pivotal member of the m6A pathway, governs a diverse and intricate array of metabolic regulatory mechanisms. Here, we present a comprehensive overview of additional metabolic processes. The cellular cycle metabolism typically encompasses two interphase intervals, together with the synthesis (S) phase and the mitotic (M) phase. Notably, tumor cells often exhibit dysregulated cell cycle progression, positioning it as a hallmark of tumorigenesis [98]. YTHDF1 assumes a significant role in this context, as it has been reported to govern the translation process of crucial regulatory factors CDK2, CDK4, and CCND1, thereby regulating the G1 to S phase transition of tumor cells in NSCLC [99]. Additionally, YTHDF1 has been found to regulate the promotion of S phase entry in breast cancer by modulating the cell cycle proteins E2, CDK2, P21, and PCNA [25]. YTHDF1 can also be modulated by METTL3, thereby regulating the translation process of the key regulatory factor CDC25B involved in the G2/M phase of cell cycle metabolism. This plays a pivotal role in the proliferation of tumor cells [100]. The association between YTHDF1 and cell cycle regulation in lung adenocarcinoma is of great significance. Lou discovered that YTHDF1 can promote the expression of cell cycle protein B1 through an m6A-dependent mechanism. Cyclin B1 is essential for the G2/M phase transition of the cell cycle. The enhanced translation of cyclin B1 has been demonstrated in this study to have a positive impact on the proliferation of KRAS/TP53 co-mutated lung adenocarcinoma cells, and is associated with poor prognosis [101].

**Figure 3:**
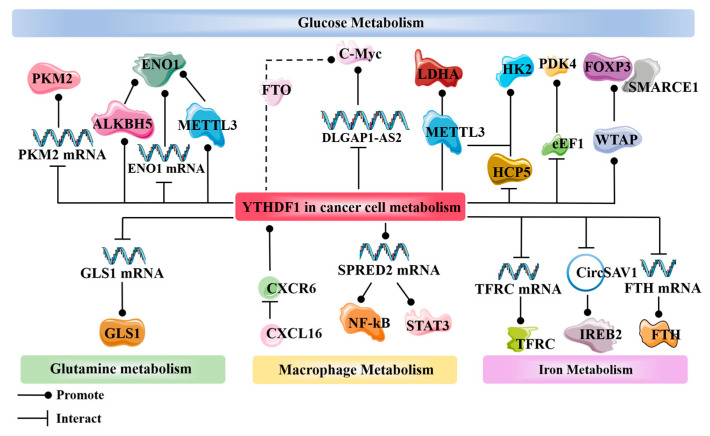
Involvement of YTHDF1 in multiple pathways of tumor metabolic reprogramming, including glucose metabolism, glutamine metabolism, macrophage metabolism, and iron metabolism. Cell cycle metabolism and lipid metabolism, as mentioned in the text, are not included.

YTHDF1 also plays a role in lipid metabolism, which is an important way for tumors to acquire energy. YTHDF1 can assist FTO in enhancing the translation of HSD17B11. The overexpression of the HSD17B11 protein promotes lipid droplet formation in esophageal cancer, thereby facilitating the development of esophageal cancer [102]. YTHDF1 also functions in hepatocellular carcinoma by promoting the translation of SLP2 through an m6A-dependent mechanism in conjunction with METTL3. SLP2 has been discovered to interact with the C-terminus of JNK2, enhancing its stability. The overexpression of JNK2 leads to the increased activity of SREBP1, and facilitates its translocation to the nucleus, ultimately resulting in enhanced de novo lipogenesis (DNL) [103] (Figure 3) (Table 2).

## 4. YTHDF1 and Cancer Therapy

Due to the increasingly in-depth research on m6A-related studies, it has gradually emerged that YTHDF1 can serve as a pivotal therapeutic target in various treatment modalities, including chemotherapy, immunotherapy, treatment with small molecule inhibitors, targeted therapy, and as a positive factor in cancer biomarker identification.

### 4.1. Chemotherapy

Chemotherapy primarily relies on the sensitivity of tumor cells to chemical drugs. YTHDF1 plays a crucial role in tumor resistance, leading to a decrease in the effectiveness of chemotherapy and impacting patient prognosis. Recent discoveries have shed light on this relationship. In gastrointestinal stromal tumors (GIST), YTHDF1 forms a complex with eEF-1 (eukaryotic elongation factor-1) and promotes the translation of MRP1 mRNA in an m6A-dependent manner. The methylation of the 5’ UTR of MRP1 mRNA plays a significant role in its translation, and increased levels of MRP1 can result in resistance to imatinib in GIST [104]. The recruitment of the YTHDF1 protein in an m6A-dependent manner with METTL3 can also promote the translation of LDHA (Lactate dehydrogenase A) mRNA in colorectal cancer. The elevated expression of LDHA leads to increased glycolysis activity in tumor cells and contributes to resistance to 5-FU chemotherapy [75]. The upregulation of GLS1 plays a role in promoting resistance to cisplatin in colon cancer cells. Moreover, GLS1 mRNA is a direct target of YTHDF1, facilitating the overexpression of GLS1 [88]. Sun et al. reported that YTHDF1 can directly target E2F8 (E2F Transcription Factor 8), with m6A writer METTL14 playing a stabilizing role in this process. E2F8 has the ability to promote cell growth and induce resistance to a range of chemotherapeutic drugs, including doxorubicin, cisplatin, and olaparib [25]. Lin discovered that YTHDF1 also plays an m6A-dependent role in the binding of METTL3 and DDX23 mRNA. DDX23 mRNA has been identified as a direct target of METTL3. The translational expression of DDX23 can promote the development of pancreatic ductal adenocarcinoma (PDAC) through the PI3K/Akt signaling pathway, and enhance resistance to gemcitabine in PDAC [105]. In bladder cancer, YTHDF1 can also induce drug resistance in tumors through this pathway. Zhu discovered that YTHDF1 can directly enhance the expression of RPN2 in the N-oligosaccharyltransferase complex in an m6A-dependent manner, along with METTL3. RPN2, through its effect on the PI3K–AKT–mTOR pathway, promotes cisplatin resistance in bladder cancer [58]. In conditions of hypoxia or low YTHDF1 expression, patients may also develop chemotherapy-induced drug resistance through YTHDF1. For instance, during platinum-based chemotherapy, the accumulation of reactive oxygen species (ROS) leads to poor clinical outcomes under the control of YTHDF1. Specifically, YTHDF1 reduces the translation efficiency of Keap1, resulting in the upregulation of Nrf2 and its downstream antioxidant factor AKR1C1 through the Keap–Nrf2–AKR1c1 axis. This upregulation contributes to the development of drug resistance in cancer cells against platinum-based therapy [99].

YTHDF1 also plays a role in the development of side effects during chemotherapy. In the case of oxaliplatin treatment, patients often experience neuropathic pain (NP). Bai discovered the mechanism through which the WNT3a/YTHDF1 pathway contributes to NP. WNT3a is capable of regulating the expression of YTHDF1 protein downstream, which promotes an increase in the levels of the associated cytokines TNF-α and IL-18, thereby inducing neuropathic pain in patients [106].

### 4.2. Immunotherapy

YTHDF1 plays a crucial role primarily in immune checkpoint inhibitor (ICI) therapy. There are several immune checkpoints involved, such as PD-1/PD-L1. ICI therapy has been proven to be effective in the treatment of tumors, but its efficacy is limited to only a fraction of patients. Understanding the specific responses to ICI treatment is essential, making it worthwhile to explore the role of YTHDF1 in this context. YTHDF1 is capable of being promoted by methionine metabolism to exert its function in methylated recognition translation, thereby facilitating the translation of immune checkpoint PD-L1 and VISTA (V-domain Ig suppressor of T cell activation). The depletion of YTHDF1 will result in the increased infiltration of CD8+ T cells and synergistically inhibit tumor proliferation in conjunction with immune checkpoint PD-1 [107]. PD-L1 mRNA is also a direct target of YTHDF1. The 3’-UTR of PD-L1 contains significant m6A modification sites (GGACA), which binding stabilizes PD-L1 mRNA and promotes PD-L1 expression. A high expression of PD-L1/PD-1 enhances tumor evasion from immune attacks [97]. In hepatocellular carcinoma associated with non-alcoholic fatty liver disease, YTHDF1 can directly target EZH2 mRNA to promote EZH2 translation, leading to the production of cytokine IL-6 in tumor cells. This subsequently triggers the infiltration of myeloid-derived suppressor cells (MDSCs) and inhibits the cytotoxic function of CD8+ T cells against tumor cells. Wang’s findings suggest that combining YTHDF1 targeting inhibition with ICI significantly enhances the therapeutic efficacy of ICI therapy [108]. In Bao’s study on colorectal cancer, similar findings were observed. YTHDF1 can directly target the subunit p65 mRNA of NF-κB, promoting its translation. This leads to the activation of the TNF/NF-κB signaling pathway and the upregulation of chemokines such as CXCL1. Interestingly, YTHDF1 also regulates the translation of CXCL1 mRNA. CXCL1 plays a crucial role in promoting the migration of MDSCs. YTHDF1, through the TNF/NF-κB signaling pathway and CXCL1, facilitates the accumulation of MDSCs, ultimately exerting immunosuppressive effects and inhibiting the function of effector T cells, leading to immune evasion. Furthermore, experimental results have shown that combining YTHDF1 knockout with anti-PD-1 immune checkpoint inhibitor therapy significantly improves treatment efficacy and reduces the tumor’s resistance to immune checkpoint inhibitors [109]. In conclusion, YTHDF1 holds tremendous potential in tumor therapy, and the combination of YTHDF1 inhibitors with ICI treatment presents a highly effective therapeutic approach.

Directly targeting and knocking out YTHDF1 can also restore anti-tumor immune activity. YTHDF1 can bind to mRNA associated with lysosome genes, promoting the translation of lysosomal proteins. These lysosomal proteins inhibit the expression of MHC-I and degrade tumor antigens, leading to a weakened ability of T cells to recognize tumors. As a result, the infiltration of CD4+ and CD8+ T cells decreases, allowing tumor cells to escape immune recognition within the human body [64]. YTHDF1 plays a similar role in GC, where its recruitment can induce a reduction in the aggregation of mature dendritic cells (DC) through immunological pathways. This decrease in DC aggregation results in the lower expression of MHCII and secretion of IL-12, thereby inhibiting the infiltration of CD4+ and CD8+ T cells. Consequently, the secretion of IFN-γ is reduced, and the JAK/STAT1 signaling pathway within tumor cells is downregulated. Ultimately, this leads to the reduced sensitivity of tumor cells to tumor immune responses, thereby facilitating immune evasion by tumor cells [110].

### 4.3. Small Molecule Inhibitors

Small molecule inhibitors are a class of bioactive compounds that possess the ability to interact with target proteins and inhibit their functional activities. Given the promoting role of YTHDF1 in various tumor types, the search for and development of small molecule inhibitors specifically targeting YTHDF1 have emerged as a crucial approach in the treatment of tumor progression. Sekulovski reported on the potential small molecule inhibitor of YTHDF1, the classic drug Niclosamide (NIC). NIC is a medication approved by the U.S. Food and Drug Administration for treating worm infections in humans. However, in recent years, it has been discovered that NIC possesses potential inhibitory effects on various tumors through multiple signaling pathways, such as Wnt, STAT3, NF-κB, and others [111]. Micaelli et al. conducted research and discovered that the small molecule ebselen can interact with the YTH domain of YTHDF proteins, consequently inhibiting their RNA binding abilities. This inhibition also applies to YTHDF1. Ebselen, an organic selenium small molecule, has been identified as a papain-like cysteine protease, and possesses properties similar to glutathione peroxidase. It is capable of forming a selenenylsulfide bond with the thiol group of cysteine residues. This small molecule has already found applications in the treatment of diabetes and neurodegenerative disorders [112]. Zou has recently discovered a remarkable compound known as SAC (salvianolic acid C). This small molecule demonstrates the exceptional ability to selectively interact directly with YTHDF1, inhibiting its RNA binding capacity and disrupting its aggregation. Consequently, this leads to a reduction in YTHDF1’s translational efficiency. SAC, derived from the traditional Chinese herb Salvia miltiorrhiza, is a natural compound renowned for its preventive effects against kidney diseases [113,114]. Hong has reported on the FDA-approved drug Tegaserod, which also serves as a potential direct targeted small molecule against YTHDF1. This remarkable medication has the ability to directly disrupt the binding between YTHDF1 and m6A-mRNA, thus inhibiting YTHDF1’s translation of cyclin E2 and subsequently slowing down cancer progression. Tegaserod, known as a 5-HT4 receptor agonist, is commonly utilized in the treatment of irritable bowel syndrome with constipation (IBS-C) [115,116]. It is foreseeable that with the continuous synthesis and development of various small molecules, coupled with the significant impact of YTHDF1 on tumors, YTHDF1 will increasingly emerge as a crucial target protein.

### 4.4. Other Therapies

The overexpression of YTHDF1 has been widely regarded as a diagnostic and prognostic biomarker in various tumors, particularly in gastric colorectal cancer, prostate cancer, cervical cancer, hepatocellular carcinoma, breast cancer, and non-small-cell lung cancer. It is associated with significantly higher expression levels and poorer prognoses in patients with these cancers [27,47,117]. Our findings also indicate that YTHDF1 plays a crucial role in the occurrence and progression of cancer. Understanding the expression patterns of YTHDF1 in various types of cancer is of great significance for clinical diagnosis and guidance.

**Figure 4:**
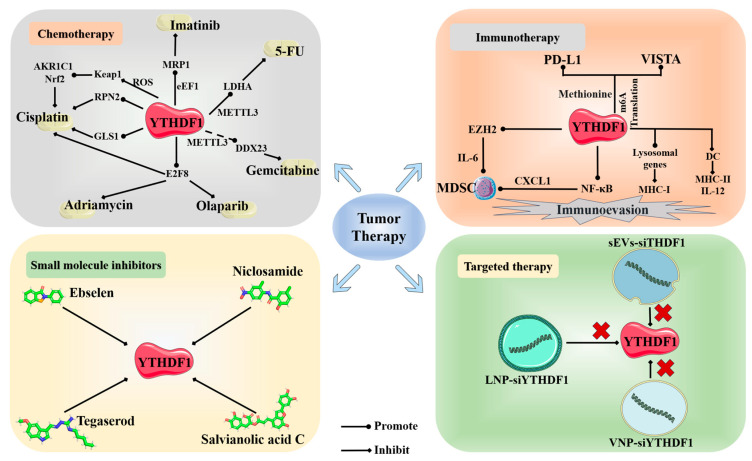
Novel therapeutic approaches targeting YTHDF1 in tumors. The 2D structures of four small molecule inhibitors targeting YTHDF1 (Ebselen, PubChem CID:3194; Niclosamide, PubChem CID:4477; Tegaserod, PubChem CID:135409453; Salvianolic acid C, PubChem CID:13991590).

Due to the significant role of YTHDF1 in cancer, targeted therapies directly aimed at YTHDF1 have been proposed. Recent reports have introduced safer and more efficient treatment approaches, such as the construction of nano-sized small extracellular vesicles (sEVs). These sEVs are engineered with high CD47 expression and modified with cyclic arginine–glycine–aspartic acid (c(RGDyC)), enabling the safe and efficient delivery of siRNA-YTHDF1. This treatment approach effectively blocks the Wnt/β-catenin pathway by deactivating the translation of frizzled7 through YTHDF1, while also triggering an anti-tumor immune response in the treatment of gastric cancer. It is worth noting that the high expression of CD47 within sEVs enhances the phagocytic activity of tumor-associated macrophages, further enhancing the therapeutic efficacy [118]. Wang utilized an FDA-approved LNP formulation to design LNP-siRNA drugs targeting YTHDF1, directly silencing its expression and thereby achieving the inhibition of cancer progression [108]. Bao developed a nanoparticle-based delivery system, specifically vesicular nanoparticle (VNP), to directly target and silence YTHDF1. Experimental evidence demonstrated that the VNP–siYTHDF1 system exhibited excellent safety and effectively suppressed tumor proliferation [109]. In conclusion, targeted therapy against YTHDF1 holds promising potential as a future clinical treatment modality. The further development of safer and more efficient carriers, along with clinical validation, is warranted (Figure 4).

## 5. Conclusions and Perspectives

YTHDF1 plays a crucial role in biological embryonic development, immune system development, and cell growth. It is also an indispensable gene in tumor initiation, tumor growth, and tumor invasion. The underlying mechanisms are intricate, with a significant portion being associated with YTHDF1’s m6A modification function. Additionally, YTHDF1 is regulated by various upstream signals, and multiple signaling pathways mediate its role in maintaining the translation expression stability of downstream target genes within cells. This ultimately contributes to the regulation of tumor growth activities.

The impact of YTHDF1 on tumors is pervasive, exerting significant influence across tumors in the digestive, respiratory, endocrine, and urogenital systems. Its role in regulating tumor initiation and progression varies depending on the distinct cellular targets it engages with. At the molecular level, YTHDF1 orchestrates a wide array of metabolic reprogramming processes within tumor cells, particularly in the realm of aerobic glycolysis where its function is indispensable, forming the bedrock of tumor genesis and invasion. The contribution of YTHDF1 in other metabolic pathways should not be disregarded either. Given the profound significance of YTHDF1 in tumors, it has been embraced as a potential therapeutic target and avenue across various treatment modalities, yielding commendable outcomes. However, the further exploration into and development of small molecule inhibitors specifically targeting YTHDF1 are still needed, aiming to identify more precise binding targets. YTHDF1 also serves as a prognostic biomarker for the majority of tumors, driving malignant progression and indicating an unfavorable prognosis. However, there are specific tumors where decreased YTHDF1 expression correlates with poor clinical outcomes, especially under platinum-based chemotherapy regimens [99]. The aforementioned highlights the substantial research potential and broad applicability of studying YTHDF1. It is imperative for us to gain a comprehensive understanding of the mechanisms through which YTHDF1 influences tumor initiation, metabolic reprogramming, and the specific regulatory mechanisms underlying related therapeutic approaches.

However, our understanding of YTHDF1 is still limited, as the m6A modification function often involves the participation of “writers”, “readers” and “erasers”. Discussing tumor development, tumor metabolism, and tumor treatment solely in relation to YTHDF1 as a “reader” protein is not comprehensive enough. The mechanisms involved typically rely on the collaborative efforts of multiple m6A modification regulators, rather than solely depending on YTHDF1. Nonetheless, these discussions do underscore the significant role played by YTHDF1 in m6A modification. In summary, as an m6A reader, YTHDF1 is an integral component in tumors, exerting a pivotal role in tumor metabolic reprogramming. It also holds vast potential for applications in tumor treatment and patient prognosis.

## Figures and Tables

**Table 1:** The involvement mechanism of YTHDF1 in various types of tumors.

**Protein**	**Mechanism**	**Function**	**Type of Cancer**	**Reference**
c-Myc	The OGT facilitates the cytoplasmic localization of YTHDF1, thereby upregulating the expression of c-Myc	Inducing tumor initiation	CRC	[30]
YY1	YTHDF1 enhances YY1 expression, and YY1 upregulates ATG4B by binding to its promoter	Dysregulating autophagy	GC	[32]
Snail	YTHDF1 interacts with eEF-2, recognizing and facilitating the expression of Snail	Inducing EMT and tumor migration	GC	[33]
SCARB1	H19 binds to YTHDF1, recognizing and promoting the translation of SCARB1 mRNA	Promoting tumor proliferation, migration, and angiogenesis	GC	[34]
c-Myc	Fn upregulates the expression of METTL3 and enhances the translation of c-Myc mRNA through YTHDF1	Promoting tumor proliferation and migration	ESCC	[36]
ANLN	YTHDF1 collaborates with METTL3 to facilitate the translation of ANLN through an m6A-dependent mechanism	Promoting bone metastasis	HCC	[39]
EGFR	YTHDF1 binds to the 3’-UTR site of EGFR mRNA and promotes translation	Promoting tumor proliferation and migration	HCC	[40]
KGA, GAC	The downregulation of YTHDF1 by B28 subsequently decreases the expression of GLS1 isoform KGA/GAC	Increasing ROS, emerging bioenergetic crisis and apoptosis	PDAC	[44]
FRAS1	METTL3-FRAS1 and YTHDF1 collaboratively regulate CDON	Facilitating tumor proliferation and colony formation	NSCLC	[47]
TGFβR2, SMAD3	YTHDF1 collaborates with YTHDF3 in the selective recognition of TGFβR2 and SMAD mRNA	Inducing EMT	NSCLC	[48]
BZLF1, BRLF1	YTHDF1, together with ZAP, DX17, and DCP2, collaboratively decaps and decreases the stability of BZLF1 mRNA and BRLF1 mRNA	Inhibiting the activation and re-infection of EBV	NPC	[51]
TUC338	YTHDF1 potentially regulates the translation stability of the lncRNA TUC338 together with METTL3	Enhancing the invasiveness of tumor cells	LC	[52]
Pten	METTL14 overexpression enhances the stability of Pten mRNA by an m6A–YTHDF1-dependent mechanism	Inhibiting tumor proliferation, migration and AKT signaling activation	RCC	[54]
ZNF677	YTHDF1 enhances the expression level of ZNF677, which significantly inhibits CDKN3 at both mRNA and protein levels	Inhibiting tumor proliferation and inducing apoptosis	RCC	[55]
RPN2	A high expression of METTL3/YTHDF1 increases the level of RPN2, thereby activating the PI3K/AKT pathway	Accelerating tumor proliferation and progression	BLCA	[58]
PLK1	The expression of YTHDF1 is activated by ELK1, thereby promoting the translation of m6A-dependent Polo-like kinase 1	Promoting tumorigenesis and metastasis	PRAD	[61]
STEAP2	Low expression of METTL3 leads to a decrease in the YTHDF1-mediated translation of STEAP2 mRNA	Enhancing tumor invasion and poor prognosis	PTC	[68]
YAP	YTHDF1 recognizes methylated YAP transcripts to promote its translation	Enhancing tumor proliferation, invasion and metastasis	OS	[69]

**Table 2:** The mechanistic role of YTHDF1 in tumor metabolic reprogramming.

Downstream Effectors	Mechanism	Metabolic Types	Types of Tumors	References
c-Myc	YTHDF1 mediates the enhancement of c-Myc mRNA stability by DLGAP1-AS2.	Glucose Metabolism	NSCLC	[73]
	The downregulation of FTO promotes the recruitment of YTHDF1 and induces the translation of c-Myc mRNA.		LUAD	[72]
LDHA	YTHDF1 is recruited by METTL3-methylated LDHA mRNA and facilitates translation.	Glucose Metabolism	CRC	[75]
PKM2	YTHDF1 enhances the translation and signaling of PKM2.	Glucose Metabolism	BLCA	[81]
ENO1	Methylated ENO1 mRNA is recognized by YTHDF1, facilitating the translation of ENO1 mRNA.	Glucose Metabolism	LUAD	[79]
HK2	METTL3 interacts with HK2 mRNA through the recognition function of YTHDF1.	Glucose Metabolism	CC	[76]
PDK4	The interaction between YTHDF1 and eEF1 enhances the translation of PDK4 mRNA.	Glucose Metabolism	CC	[76]
HK2, HCP5	The direct interaction between YTHDF1 and HCP5 amplifies the translation of HK2 mRNA.	Glucose Metabolism	ESCC	[77]
FOXP3	The collaborative action of YTHDF1 and WTAP stabilizes FOXP3 mRNA, enabling its interaction with SMARCE1 and activation.	Glucose Metabolism	CRC	[80]
GLS1	YTHDF1 selectively binds to the 3’ UTR of GLS1 mRNA and enhances translation.	Glutamine metabolism	CRC	[88]
SPRED2	YTHDF1 mediates the translation of SPRED2, thereby activating NF-kB and STAT3.	Macrophage Metabolism	LUNG	[92]
TFRC	YTHDF1 selectively binds to both the 3’UTR and 5’UTR of TRFC mRNA, thereby enhancing translation.	Iron Metabolism	HPSCC	[94]
IREB2	YTHDF1 interacts with CircSAV1 to form a ternary complex with IREB2 mRNA, thereby promoting the translation of IREB2.	Iron Metabolism	COPD	[95]
FTH	YTHDF1 facilitates the translation of FTH, thereby accelerating the transport of free iron.	Iron Metabolism	LUAD	[96]
CDC25B	YTHDF1 is modulated by METTL3 to regulate the translation of CDC25B.	Cellular Cycle Metabolism	CC	[100]
Cyclin B1	YTHDF1 promotes translation of Cyclin B1.	Cellular Cycle Metabolism	LUAD	[101]
HSD17B11	YTHDF1 decreases the translation efficiency of HSD17B11.	Lipid metabolism	ESCC	[102]
SLP2	YTHDF1 facilitates the translation of SLP2 in an m6A-dependent manner, which promotes the interaction between SLP2 and the C-terminus of JNK2, thereby enhancing the activity of SREBP1.	Lipid metabolism	HCC	[103]

## Data Availability

The dataset generated and/or analyzed during the current study is available in the RCSB Protein Data Bank (RCSB PDB) (https://www.rcsb.org/, accessed on 15 November 2023), GeneExpression Profiling Interactive Analysis (GEPIA) (http://gepia.cancer-pku.cn/, accessed on 15 November 2023), PubChem at the National Institutes of Health (NIH) (https://pubchem.ncbi.nlm.nih.gov/, accessed on 15 November 2023) and cBioPortal database (https://www.cbioportal.org/, accessed on 15 November 2023).
